# Neurovascular coupling response to cognitive examination in healthy controls: a multivariate analysis

**DOI:** 10.14814/phy2.13803

**Published:** 2018-07-23

**Authors:** Lucy Beishon, Claire A. L. Williams, Thompson G. Robinson, Victoria J. Haunton, Ronney B. Panerai

**Affiliations:** ^1^ Department of Cardiovascular Sciences University of Leicester Leicester United Kingdom; ^2^ NIHR Leicester Biomedical Research Centre University of Leicester Leicester United Kingdom

**Keywords:** Addenbrooke's cognitive examination, cerebral blood flow, cognitive tasks, neurovascular coupling, transcranial doppler ultrasonography

## Abstract

Cognitive testing with transcranial Doppler ultrasonography (TCD) has been used to assess neurovascular coupling (NVC), but few studies address its multiple contributions. Subcomponent analysis considers the relative myogenic (resistance area product, RAP) and metabolic (critical closing pressure (CrCP)) contributors. The aim of this study was to investigate the changes in subcomponents that occur with cognitive stimulation with the Addenbrooke's Cognitive Examination (ACE‐III) in healthy controls. Healthy volunteers underwent continuous recording of bilateral TCD, heart rate (HR, three‐lead ECG), end‐tidal CO_2_ (ETCO_2_, capnography), and mean arterial pressure (MAP, Finometer). The study comprised a 5‐min baseline recording, followed by all 20 paradigms from the ACE‐III. The cerebral blood flow velocity (CBFv) response was decomposed into the relative contributions (subcomponents); *V*_BP_ (MAP), *V*_CrCP_ (CrCP), and *V*_RAP_ (RAP). Data are presented as peak population normalized mean changes from baseline, and median area under the curve (AUC). Forty bilateral datasets were obtained (27 female, 37 right hand dominant). *V*_BP_ increased at task initiation in all paradigms but differed between tasks (range (SD): 4.06 (8.92)–16.04 (12.23) %, *P* < 0.05). HR, but not ETCO_2_, also differed significantly (*P* < 0.05). Changes in *V*_RAP_ reflected changes in MAP, but in some paradigms atypical responses were seen. *V*_CrCP_ AUC varied significantly within paradigm sections (range [SD]: 18.4 [24.17] to 244.21 [243.21] %*s, *P* < 0.05). All paradigms demonstrated changes in subcomponents with cognitive stimulation, and can be ranked based on their relative presumed metabolic demand. The integrity of NVC requires further investigation in patient populations.

## Introduction

As the population ages, the world prevalence of dementia is expected to reach 131 million by 2050, with limited diagnostics and therapeutics presently available (Prince et al. 2015; Alzheimer's‐society 2016). Identifying an early, sensitive marker that can distinguish dementia from normal aging is of paramount importance to facilitate early intervention with novel therapeutics (Alzheimer's‐society 2016). Cerebral hemodynamics are one such marker, with several recent reviews and a meta‐analysis demonstrating impaired cerebral perfusion in both Alzheimer's disease (AD) and vascular dementia (VaD), with the ability to reliably discriminate between the two (Keage et al. 2012; Sabayan et al. 2012). Importantly, similar markers are now emerging for mild cognitive impairment (MCI), characterized by early cognitive decline with retained functional independence, with the attractive potential for therapeutic intervention (Hays et al. 2016).

Transcranial Doppler ultrasonography (TCD) is a noninvasive technique which uses ultrasound to measure cerebral blood flow velocity (CBFv) in the intracranial arteries, including the middle cerebral artery (MCA) (van Beek et al. 2008). It is advantaged by its portability, acceptability to patients, lack of ionizing radiation, and relative ease with which to train operators (van Beek et al. 2008; Gommer 2013). Compared to other available modalities, (positron and single photon emission tomography, and functional magnetic resonance imaging), it is cheaper and more widely accessible, with better temporal resolution, albeit with reduced spatial discrimination (van Beek et al. 2008; Tomek et al. 2014). TCD can be utilized to measure neurovascular coupling (NVC) by recording CBFv during cognitive stimulation (Stroobant and Vingerhoets 2000). In a recent publication by this group, the Addenbrooke's cognitive examination (ACE‐III) resulted in increased CBFv in tasks of attention, memory, visuospatial, and language domains (Beishon et al. 2017b; Williams et al. 2017). The ACE‐III is a well‐validated diagnostic instrument, used routinely in clinical practice for the diagnosis of both dementia and MCI, with better sensitivity than the widely used Mini Mental State Examination (Mioshi et al. 2006; Velayudhan et al. 2014).

Cerebral autoregulation is the process by which cerebral blood flow (CBF) is maintained relatively constant despite fluctuations in arterial blood pressure (Aaslid et al. 1989; Panerai et al. 2005; Gommer 2013). NVC describes the mechanism by which CBF is increased to meet the rising metabolic demands of the cerebral cortex during times of increased activity (Stroobant and Vingerhoets 2000; Phillips et al. 2016). There are several components which mediate this process (Gommer 2013; Phillips et al. 2016); the metabolic response increases CBF through increased oxygen demand, central command, the production of metabolites, or rising CO_2_; the myogenic as a result of vessel smooth muscle contraction in response to transmural pressure changes; and neurogenic as a function of the autonomic nervous control of vascular smooth muscle (Gommer 2013; Phillips et al. 2016). Cerebrovascular resistance index (CVRi) is one of the more widely reported parameters (Gommer et al. 2012; Liu et al. 2014) in studies of functional TCD and represents the resistance within the intracranial small vessels (Panerai et al. 2005; Phillips et al. 2016). The use of CVRi as a marker of vessel resistance is limited by the assumption that flow within the vessel only reaches zero as perfusion pressure reaches zero, which has been contradicted by a number of studies (Aaslid et al. 2003; Panerai 2003; Panerai et al. 2005). A large number of studies have concluded that a two‐parameter model provides a more accurate representation of the instantaneous pressure–velocity relationship (Panerai et al. 2005). Critical closing pressure (CrCP) is one of the subcomponents and describes the pressure at which flow in the vessel reaches zero, which can be measured through extrapolation of the CBFv‐ABP regression line (Aaslid et al. 2003; Panerai et al. 2005). Secondly, RAP, describes the change in CBFv for a given change in perfusion pressure, and can be derived from the inverse of the CBFv‐ABP regression line (Panerai 2003; Panerai et al. 2005). CrCP is thought to represent the slower, metabolic response to CBF regulation (Panerai et al. 2005, 2012a; Salinet et al. 2013b; van Veen et al. 2015; Phillips et al. 2016), whereas RAP has been associated with the faster myogenic response of autoregulation (Panerai et al. 2005, 2012a; Salinet et al. 2013b; van Veen et al. 2015; Phillips et al. 2016). This hypothesis is supported by a number of studies showing that CrCP is affected by the partial pressure of CO_2_, and hyperemia, (Aaslid et al. 2003; Panerai 2003; Salinet et al. 2013b), and RAP as a reflection of changes in mean arterial pressure (MAP) in both healthy and diseased individuals (Panerai et al. 2005; Salinet et al. 2013b; van Veen et al. 2015).

In addition to the subcomponents described above, it is important to consider the effects of end‐tidal CO_2_ (ETCO_2_), MAP, and heart rate (HR) that occur during neuroactivation and which could contribute significantly to changes in CBFv (Stroobant and Vingerhoets 2000; Panerai et al. 2005; Gommer et al. 2014). Significant rises in HR and MAP at task initiation have been demonstrated in a number of neuroactivation studies (Moody et al. 2005; Panerai et al. 2005; Matteis et al. 2009; Salinet et al. 2012a, 2013b), but not consistently (Matteis et al. 2001; Sorond et al. 2008). This may be due to the varying complexity or sympathetic response induced by different paradigms (Salinet et al. 2013a). Using multivariate modeling, Panerai et al. (2012b) demonstrated that approximately 20% of the CBFv response to motor stimulation is due to MAP, and ETCO_2_ was accountable for <10% of the CBFv response to cognitive stimulation (Panerai et al. 2012a). ETCO_2_ is known to affect CBF (Markwalder et al. 1984), but studies thus far have shown little convincing evidence for a role in NVC in task activation (Matteis et al. 2009; Panerai et al. 2012a; Salinet et al. 2012a). However, previous studies have used a limited range of cognitive paradigms to evoke changes in CBFv (Droste et al. 1989; Moody et al. 2005; Sorond et al. 2008; Matteis et al. 2009), and those requiring more verbalization, or that induce breath holding, may result in significantly different levels of ETCO_2_, which might require further consideration (Droste et al. 1989).

While there have been several studies examining NVC in healthy volunteers, few have performed a subcomponent analysis of these data (Aaslid 1987; Vingerhoets and Stroobant 1999; Stroobant and Vingerhoets 2000; Sorond et al. 2008). In order to determine that changes in CBFv are not simply a reflection of the changes in MAP, analysis of the subcomponents of the instantaneous pressure–velocity relationship would provide evidence for cerebral autoregulation occurring at the level of the cerebral vascular bed (Aaslid et al. 2003; Panerai et al. 2005; Phillips et al. 2016). The peak and area under the curve responses in CBFv for these data have been published previously (Beishon et al. 2017b; Williams et al. 2017). Therefore, the aim of this study was to analyze the subcomponents (MAP, RAP, and CrCP) of the hemodynamic responses to cognitive stimulation using the ACE‐III assessment as well as HR and ETCO_2_, and identify the relative contributions of the presumed metabolic and myogenic components of this response.

## Methods

This was a cross‐sectional study undertaken over a period of 4 months (Feb–May 2016) at the University of Leicester, UK. Healthy volunteers were recruited by poster advertisement or email invitation as either members of faculty or students. Inclusion criteria were, aged over 18 years and willingness to participate. Exclusion criteria were pregnancy, planning pregnancy, or lactating. The study had University of Leicester ethical approval (ref: 5355‐vjh12‐cardiovascularsciences) and all volunteers provided informed consent prior to study inclusion. Volunteers were requested to avoid caffeine, nicotine, alcohol, strenuous exercise, and large meals for at least 4 h prior to recordings.

All recordings were performed in a quiet, temperature controlled (24°C) laboratory. First, data were collected on baseline demographics, medical comorbidities, and medication use. Handedness was assessed using the Edinburgh Handedness Inventory (Oldfield 1971), with both right‐ and left‐handed individuals included. All volunteers underwent bilateral TCD (secured using a head frame) of the middle cerebral artery (MCA) using Viasys Companion III. In addition, continuous measurements were taken for beat‐to‐beat blood pressure (BP, arterial volume clamping on the nondominant hand, Finometer, Finapres Medical Systems; Amsterdam, the Netherlands), HR (three‐lead electrocardiogram), and ETCO_2_ (capnography by Salter Labs, ref. 4000; Capnocheck Plus). Signals were sampled at 500 samples per second and stored in the PHYSIDAS data acquisition system. During recordings, the Physiocal function of the Finometer was turned off to prevent contamination of the data but was turned on in between recordings to allow for calibration. The total protocol duration was approximately 1.5 h. Each participant first underwent a 5‐min baseline recording during which they were instructed to rest quietly with their eyes open. This was followed by all tasks from the ACE‐III which were divided into three domains; the ‘A’ domain included paradigms from the attention (*n* = 4), memory (*n* = 3), and fluency (*n* = 2) domains; the ‘B’ domain comprised of six language paradigms; and the ‘C’ domain comprised of visuospatial (*n* = 4) and final memory (*n* = 1) paradigms. The ACE‐III was performed in the standard order that it would be undertaken clinically. Table 1 details the individual tasks, subclassified by domain. Each domain of the ACE‐III began with a 1‐min period of rest, and each task was separated by 30 sec of rest. An event‐marker was used to note question timings, and brachial BP (UA767 BP monitor) of the dominant arm was measured prior to each recording for manual calibration of the Finometer. Data analysis was performed offline using software previously developed by this group (Panerai et al. 2005; Salinet et al. 2013a,b). Data were examined visually for large nonphysiological spikes, which were removed by linear interpolation. Smaller spikes in the CBFv signal were removed with a median filter and all signals were low‐pass filtered with a zero phase Butterworth filter with a cut‐off frequency of 20 Hz. Data from three‐lead ECG recordings were used to determine the R‐R interval in order to derive mean beat‐to‐beat CBFv, HR, and ETCO_2_. For each cardiac cycle, estimates of CrCP and RAP were obtained with the first harmonic method (Panerai 2003; Panerai et al. 2011). In order to develop a uniform time base, all beat‐to‐beat‐derived parameters underwent third‐order polynomial interpolation and then resampling at 5 Hz.

**Table 1:** Paradigms from the ACE‐III used to elicit changes in CBFv, classified by domain

Paradigm	Domain	Detail
A domain		
A1	Attention	Orientation to time (day/date/month/year/season)
A2	Attention	Orientation to space (floor/building/town/county/country)
A3	Attention	Repeat and remember three words (lemon/key/ball)
A4	Attention	Subtract serial sevens from 100
A5	Memory	Recall the three words learnt earlier (A3: lemon/key/ball)
A6	Fluency	Naming words beginning with “P” in 1 min
A7	Fluency	Naming animals in 1 min
A8	Memory	Learn and remember a name and address
A9	Memory	Names of current and previous UK prime minister and US president
B domain		
B1	Language	Following verbal instructions
B2	Language	Writing two sentences
B3	Language	Repeating words and phrases aloud
B4	Language	Naming objects
B5	Language	Linking objects with statements
B6	Language	Reading words aloud
C domain		
C1	Visuospatial	Drawing an infinity diagram and three‐dimensional cube
C2	Visuospatial	Drawing a clock face and correctly positioning the hands to a given time
C3	Visuospatial	Counting number of dots
C4	Visuospatial	Recognizing obscured words
C5	Memory	Recalling the previously learnt name and address (A8)

### Subcomponent analysis

The methods used to describe the CBFv response in terms of subcomponents has been reported in detail previously (14). In summary, at rest, baseline CBFv (V_0_) can be described in terms of MAP (BP_0_), CrCP (C_0_), and RAP (*R*_0_) as outlined in the following equation (14, 23):

(1) $$V_{0}=\frac{BP_{0}-C_{0}}{R_{0}}$$

During task activation, the change in CBFv (∆*V*) can be described as the sum of the changes in subcomponents in addition to the resting values (14, 23):

(2) $$\left(V_{0}+\Delta V\right)=\frac{\left(BP_{0}+\Delta BP\right)-\left(C_{0}+\Delta C\right)}{R_{0}+\Delta R}$$

Therefore, the change in CBFv can be determined from the sum of its subcomponents, corresponding to the contributions of MAP, CrCP, and RAP. Normalizing the CBFV change in relative (or %) values as Δ*v* = Δ*V*/*V*_0_, the corresponding relative contributions of MAP, CrCP, and RAP can be expressed as *V*_Bp_, *V*_CrCP_, and *V*_RAP_, respectively (14, 23):

(3) $$\Delta v=V_{\mathrm{BP}}+V_{\mathrm{crcp}}+V_{\mathrm{RAP}}$$

If Δ*R* << *R*_0_, the subcomponents will be given by:

(4) $$V_{\mathrm{BP}}=\frac{\Delta \mathrm{BP}}{V_{0}R_{0}}$$

(5) $$V_{\mathrm{crcp}}=-\frac{\Delta C}{V_{0}R_{0}}$$

(6) $$V_{\mathrm{RAP}}=\frac{\Delta R}{R_{0}}$$

Due to the negative signs in Equation (5), (6), falling CrCP is represented by a rise in its relative subcomponent (*V*_CrCP_), and reflects a positive contribution (increase) to the CBFv response. The same is true of RAP, where rising RAP is represented by a fall in the relative subcomponent (*V*_RAP_) and therefore reflects a negative contribution (decrease) to the CBFv response (14, 23).

### Statistical analysis

Data are presented as population averaged peak change in MAP, HR, and ETCO_2,_ normalized to a 20‐sec baseline period prior to task initiation. The peak response was calculated as the maximal percentage change between 25 and 30 sec (T2), where task initiation occurred at 20 sec. In a preliminary analysis, *V*_CrCP_ had the greatest normalized mean percentage from baseline, and, given that *V*_CrCP_ is thought to reflect the metabolic component of cerebral autoregulation, this was chosen as the focus for more detailed analysis. Furthermore, given the number of cognitive tasks used in this protocol and the number of variables it was not practical to undertake this analysis for all parameters reported here. The change in peak and area under the curve data for CBFv in response to task activation with the ACE‐III have been published previously (Williams et al. 2017)(ref main paper). Individual changes in *V*_CrCP_ were variable in duration and peak. We therefore present population averaged area under the curve for *V*_CrCP_ for each task (AUC *V*_CrCP_). The time interval for the AUC analysis varied depending on the task or the hemisphere, and therefore individual time intervals were used for each task in each hemisphere, based on the population averaged curves. Data were nonparametric in distribution, and could not be normalized by log transformation. Data are therefore presented as mean [standard deviation] for parametric (MAP, HR, ETCO_2_) and median [IQR] for nonparametric continuous variables (*V*_CrCP_ AUC). Statistical analysis was by repeated measures analysis of variance (ANOVA) for parametric data, and the Friedman test for related samples, nonparametric data. Repeated paired Wilcoxon tests with Bonferroni correction for repeated measures were used to detect the significance between dominant and nondominant hemispheres. Analyses were considered significant if *P* < 0.05. Statistical analysis was performed using Statistica Version 13 software for Windows.

## Results

Forty‐eight volunteers were recruited to the study, of whom 40 participants had good quality bilateral data suitable for analysis. Reasons for exclusion of the eight participants were inadequate windows (*n* = 1), insufficient data quality or Finometer drift (*n* = 6), failure of equipment or technical fault (*n* = 1). The 40 participants included in the study were relatively young (median age 31 years [IQR: 22–52]), and the majority were female (*n* = 27), right hand dominant (*n* = 37), and Caucasian (*n* = 36). None of the included participants were current smokers. The mean ACE‐III score for volunteers was high (98), with no participant falling below the cut‐off for MCI and dementia.

### Cardiovascular parameters

The *V*_BP_ increased at task initiation for all paradigms (25–30 sec), and HR increased for 18 paradigms (mean *V*_BP_ range: 1.2–16.04% (7.22–12.23), HR range: 71.9–78.9 bpm (8.13–10.13)), Table 2. The largest increase was seen with the animal naming task (A7) (mean *V*_BP_ increase: 16.04% (12.23)), whereas recognizing obscured words (C4) resulted in the smallest rise (mean increase: 1.2% (9.25)), Table 2. Changes in *V*_BP_ and HR varied significantly across paradigms within the A and C domains (*P* < 0.05), Table 2. ETCO_2_ did not vary significantly across the A, B, and C paradigms, Table 2.

**Table 2:** Peak normalized population mean changes from a 20‐sec baseline period for all paradigms

Paradigm	*n*	*V*_RAP_ (%)	*V*_CrCP_ (%)	*V*_BP_ (%)	HR (%)	ETCO_2_ (%)
A domain						
A1	40	−1.0 (7.8)	2.6 (6.3)	8.1 (8.8)	5.9 (7.4)	0.1 (2.6)
A2	40	−0.8 (7.2)	1.8 (6.8)	8.7 (8.4)	1.6 (5.8)	−0.3 (4.2)
A3	40	−1.7 (9.6)	2.2 (5.2)	9.5 (7.2)	2.8 (7.5)	1.9 (5.7)
A4	40	−3.2 (7.7)	3.1 (5.0)	10.4 (8.8)	7.0 (9.0)	−0.6 (3.8)
A5	40	−4.8 (10.0)	1.0 (7.8)	8.3 (10.8)	−0.1 (6.2)	0.6 (4.2)
A6	40	−4.5 (8.8)	2.2 (6.5)	10.4 (8.8)	7.6 (7.8)	0.1 (2.9)
A7	40	−10.6 (11.9)	1.9 (5.8)	16.0 (12.2)	7.2 (7.1)	0.1 (7.8)
A8	40	−4.3 (10.7)	2.0 (5.5)	7.2 (10.0)	1.7 (8.9)	−1.1 (9.0)
A9	40	−3.7 (9.7)	2.1 (7.5)	9.6 (9.7)	2.3 (5.8)	2.4 (7.3)
*P* value		**<0.005**	0.89	**0.001**	**<0.005**	0.19
B domain						
B1	40	1.2 (8.4)	3.4 (7.2)	5.9 (8.5)	1.7 (5.5)	0.3 (3.6)
B2	40	0.3 (8.8)	2.7 (6.7)	8.6 (9.3)	2.8 (7.8)	1.1 (4.7)
B3	40	−2.3 (8.1)	−0.0 (6.0)	9.6 (8.6)	−0.7 (6.4)	1.4 (5.8)
B4	40	−1.8 (9.2)	3.2 (6.4)	7.6 (9.6)	1.1 (8.1)	−0.2 (4.9)
B5	40	−0.1 (8.0)	4.6 (6.4)	6.2 (9.2)	−0.8 (5.8)	1.0 (8.4)
B6	40	2.2 (7.7)	−0.1 (6.1)	4.1 (8.9)	0.7 (6.7)	1.7 (3.9)
*P* value		0.28	**0.027**	**0.03**	0.1	0.53
C domain						
C1	40	−0.7 (11.5)	5.8 (6.1)	5.3 (9.2)	5.1 (7.1)	−0.1 (3.4)
C2	40	−4.1 (11.0)	6.0 (9.4)	8.2 (11.5)	2.5 (5.5)	0.6 (6.3)
C3	40	−0.7 (8.8)	4.4 (6.3)	3.1 (9.3)	−0.3 (6.0)	1.0 (7.9)
C4	40	1.6 (9.4)	2.9 (5.0)	1.2 (9.3)	0.3 (4.4)	1.1 (6.6)
C5	40	−3.1 (10.0)	3.2 (8.2)	10.1 (10.1)	3.5 (8.0)	−0.9 (5.6)
*P* value		**0.025**	0.13	**<0.005**	**<0.005**	0.62

The normalized (%) change in CBFv was decomposed in its subcomponents due to parallel changes in MAP (*V*_BP_), CrCP (*V*_CrCP_), and RAP (*V*_RAP_). See Equations [1‐6]. Data are for the dominant hemisphere only. Data are presented as mean (standard deviation) percentage change. The peak value was taken at 25–30 sec, where task initiation occurred at 20 sec. Significance testing is by repeated measures ANOVA. Bold values represent statistically significant values.

### *V*_CrCP_ and *V*_RAP_ at T2 (25–30 sec)

**Figure 1:**
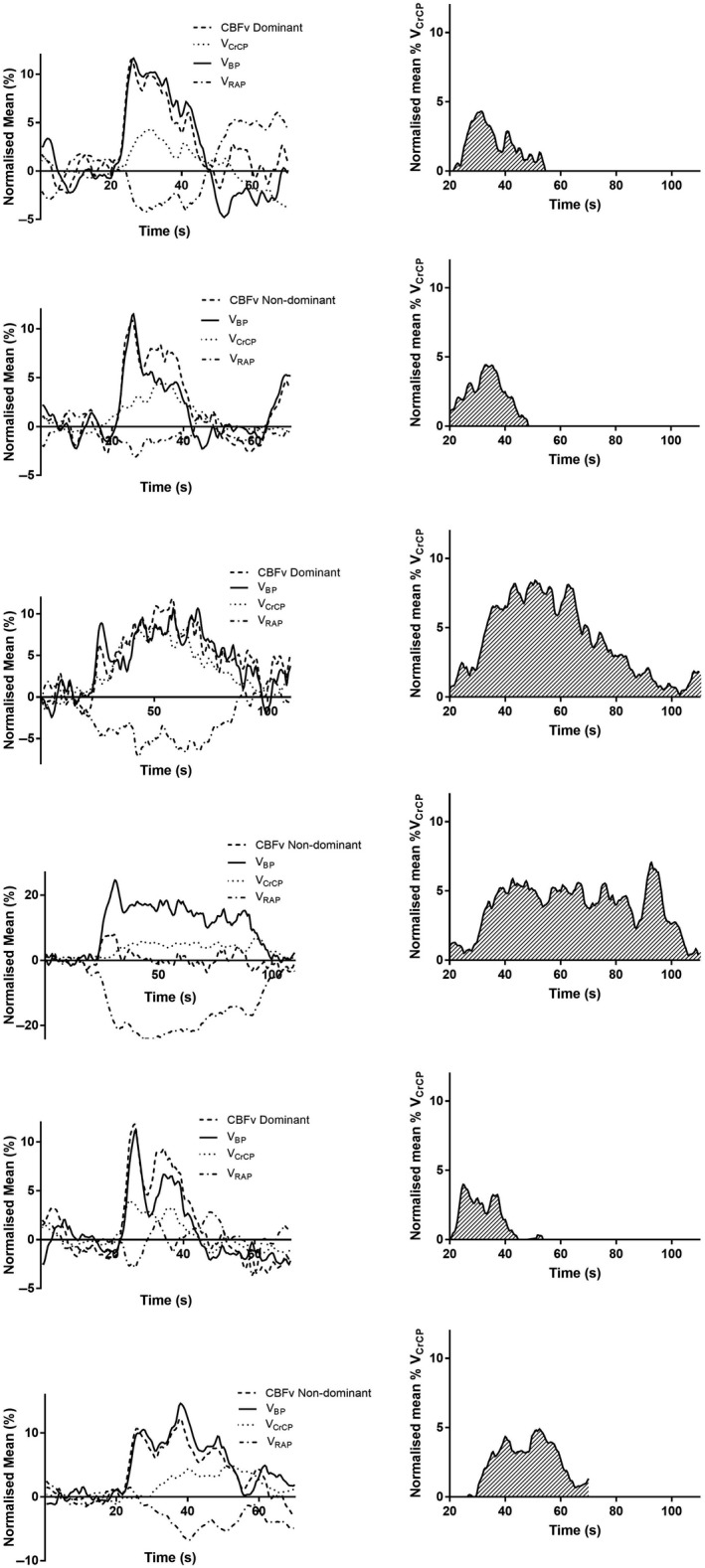
Normalized population mean (*n* = 40) changes in CBFv and its subcomponents for selected paradigms from the ACE‐III. Left and right panels represent the dominant and nondominant hemispheres, respectively. Smaller figures show the AUC for *V*_CrCP_ for each paradigm. Solid line = *V*_BP_, dashed line = CBFv, dotted line = *V*_CrCP_, dashed and dotted line = *V*_RAP_. A4, B4 (dominant) and A1, B3 (nondominant) represent the smallest *V*_CrCP_ AUC for the A and B domains, and A8 (dominant), and A7 (nondominant) represent that largest VCrCP AUC for the A domain. CBF, cerebral blood flow velocity.

**Figure 2:**
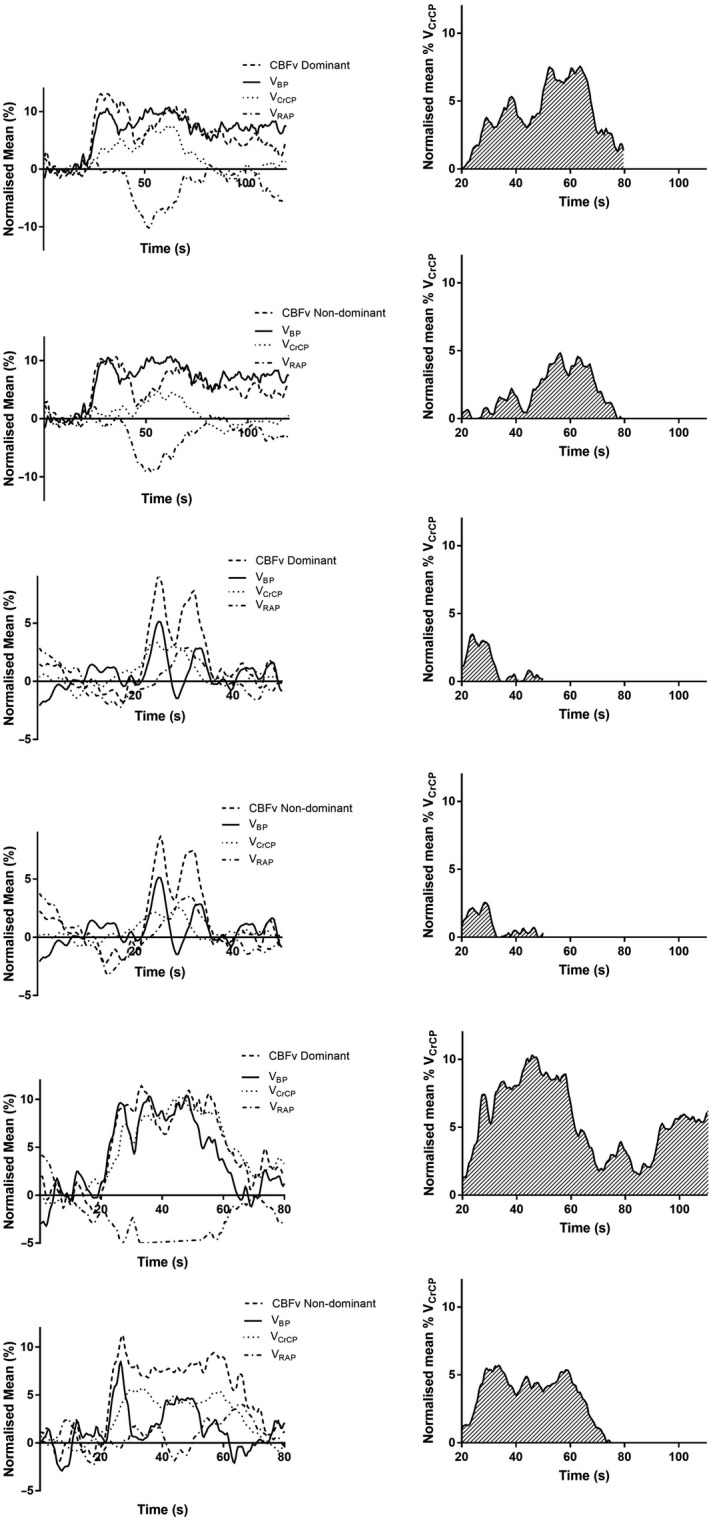
Normalized population mean (*n* = 40) changes in the subcomponents for selected paradigms form the ACE‐III. Left and right panels represent the dominant and nondominant hemispheres, respectively. Smaller figures show the AUC for VCrCP for each paradigm. Solid line = *V*_BP_, dashed line = CBFv, dotted line = *V*_CrCP_, dashed and dotted line = *V*_RAP_. B2, C2 (dominant), B2, C1 (nondominant) represent the largest *V*_CrCP_ AUC for the B and C domains, C4 (dominant and nondominant), represent the smallest *V*_CrCP_ AUC for the C domain. CBF, cerebral blood flow velocity.

**Figure 3:**
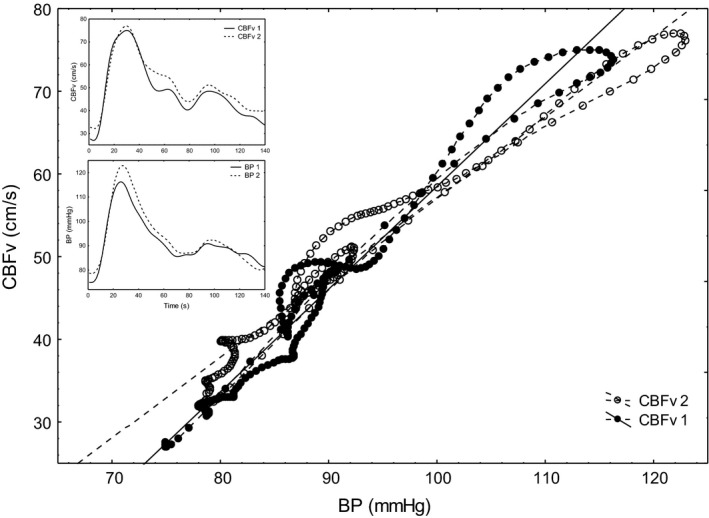
Representative velocity–pressure curves from a 21‐year‐old female participant for the A7 paradigm. The original continuous recording for the corresponding cardiac cycles in shown in the inset. The point at which the regression line reaches zero on the CBFv (y axis), for CBFv1 (solid line), and CBFv2 (interrupted line) represents CrCP (CBFv1: 53.69, CBFv2: 51.20). RAP for CBFv 1: 7.88, CBFv 2: 8.51. The graph demonstrates the typical change in CrCP and RAP after task initiation at 20 sec. CBF, cerebral blood flow velocity

Nineteen of the 20 paradigms produced rises in *V*_CrCP_, but to differing extents, (mean range: −0.1–5.99% (5.02–9.43)). The rise in *V*_CrCP_ occurred more gradually than that seen with CBFv and MAP, tending to peak later in the response, Figures 1, 2. Conversely, not all paradigms produced decreases in *V*_RAP_ (mean range: −10.58–2.18% (7.22–11.92)), Table 2, Figures 1, 2. Figures 1, 2 demonstrate the temporal patterns in the subcomponent responses to the paradigms which produced the largest and smallest changes in *V*_CrCP_ AUC for each section of the ACE‐III. The majority of paradigms demonstrated typical *V*_CrCP_ (increasing) and *V*_RAP_ (decreasing) responses to cognitive stimulation. However, B4, C4, and C1 paradigms demonstrate atypical changes, with paradoxical rises in *V*_RAP_ and *V*_CrCP_. Peak *V*_RAP_ response differed significantly within the B and C paradigm domains (*P* < 0.05), but peak *V*_CrCP_ was only significantly different within the B domain (*P* = 0.027). This may in part be due to the 25–30‐sec time period, where the majority of *V*_CrCP_ responses peak later, and therefore AUC may be a more reliable measure. Figure 3 shows representative instantaneous velocity–pressure relationships from a 21‐year‐old female participant, from one beat at 20 sec (CBFv1 shaded markers), and at 40–50 sec (CBFv2, clear makers), showing the changes in CrCP and RAP taking place following neural stimulation.

### V_CrCP_ AUC analysis

*V*_CrCP_ AUC varied significantly across all paradigms, within the A, B, and C domains (*P* < 0.05). Paradigms also produced differences between hemispheres, Table 3. Table 3 shows the AUC for *V*_CrCP_ for each paradigm, ordered relative to the dominant hemisphere. Within the A paradigms (dominant hemisphere), the smallest AUC was seen with A4 (serial subtraction), (39.36%*s [79.39], 20–55 sec), Table 3, and the largest with A8 (learning a name and address) (244.21%*s [243.21], 20–105 sec), Table 3. In the nondominant hemisphere, the smallest AUC was seen with A1 (orientation to time) (18.4%*s [24.17], 20–50 sec), and the largest with A7 (naming animals) (199.85%*s [302.71], 20–110 sec).

**Table 3:** V_CrCP_ AUC values for each paradigm and the time interval used to calculate the AUC, based in the duration of the response

Paradigm	*N*	Dominant		Nondominant		*P* value
		AUC (%*s)	Time interval (s)	AUC (%*s)	Time interval (s)	
A4	40	39.4 (79.4)	20–55	40.3 (36.6)	20–5	1.0
A1	40	41.9 (38.8)	20–45	18.4 (24.2)	20–50	1.0
A5	40	44.4 (58.7)	20–50	40.5 (60.0)	25–50	0.4
A2	40	46.7 (54.5)	20–52	38.1 (81.09)	30–50	**<0.005**
A3	40	55.8 (78.4)	20–55	31.8 (49.07)	20–55	0.6
A9	40	91.6 (162.9)	20–85	54.9 (106.05)	20–85	0.07
A6	40	119.7 (170.9)	20–65	131.8 (218.97)	20–70	**<0.005**
A7	40	148.0 (275.7)	20–110	199.9 (302.71)	20–110	1.0
A8	40	244.2 (243.1)	20–105	85.3 (74.49)	20–110	1.0
Overall						***P*** ** < 0.005**
B4	40	30.2 (47.9)	20–45	47.8 (115.6)	20–45	1.0
B6	40	40.1 (137.1)	20–75	44.4 (79.1)	20–70	0.05
B1	40	56.6 (119.6)	20–70	67.2 (108.5)	25–65	**0.048**
B3	40	66.0 (101.45)	25–60	27.75 (42.8)	30–70	1.0
B5	40	90.8 (119.7)	20–70	61.0 (96.85)	20–70	0.1
B2	40	111.3 (211.8)	20–80	69.9 (135.2)	20–80	**<0.005**
Overall						***P*** ** < 0.005**
C4	40	14.9 (37.8)	20–35	15.8 (25.9)	20–35	1.0
C3	40	47.1 (81.2)	20–52	37.1 (67.2)	20–50	0.2
C5	40	56.9 (88.4)	20–70	54.7 (52.0)	20–65	0.4
C1	40	125.3 (158.9)	20–75	112.2 (204.9)	20–80	1.0
C2	40	161.0 (247.6)	25–65	100.5 (167.2)	20–75	1.0
Overall						***P*** ** < 0.005**

Data are nonparametric and presented as median (IQR). Paradigms are in order of smallest to largest AUC by dominant hemisphere, within each paradigm section. Bold values represent statistically significant values.

In the B section, dominant hemisphere, the smallest AUC was with B4 (recognizing objects) (30.24%*s [47.85], 20–45 sec), and the largest with B2 (writing sentences) (111.25%*s [211.84], 20–80 sec), Table 3. In the nondominant hemisphere, the smallest AUC was seen with B3 (repeating words and phrases aloud) (27.75 [42.76], 30–70 sec), and the largest with B2 (writing sentences) (69.85%*s [135.19] 20–80 sec), Table 2.

In the C domain, the smallest AUC was produced by C4 (recognizing obscured words), (dominant: 14.92%*s [37.78], 20–35 sec; nondominant: 15.78%*s [25.86], 20–35 sec). The C paradigms demonstrated greater similarities between the dominant and nondominant hemispheres, than the A or B group paradigms. The largest change in the dominant hemisphere was with C2, clock drawing, (161.01%*s [247.64], 25–65 sec), and in the nondominant hemisphere with C1 (construction of a 3‐D cube and infinity diagram), (112.19%*s [204.86] 20–80 sec).

## Discussion

### Summary of results

To our knowledge, this is the first study to perform a subcomponent analysis of the hemodynamic responses to a complete cognitive assessment battery (ACE‐III). A limited number of studies have undertaken subcomponent analysis within NVC (Panerai et al. 2005, 2012a; Salinet et al. 2013a,b), but none to such an extensive range of cognitive tasks or quantifying the contribution of *V*_CrCP_ using the AUC. Here, we demonstrate that all 20 paradigms of the ACE‐III resulted in changes in *V*_BP_, HR, *V*_CrCP_, and *V*_RAP_, in both temporal pattern and peak effect. There was significant variation between paradigms, and between hemispheres, in these responses, and paradigms can therefore be ranked according to the degree of their presumed metabolic response. Furthermore, while some paradigms showed a typical rise in *V*_CrCP_, and associated fall in *V*_RAP_, others had unexpected and paradoxical rises in *V*_RAP_ (B4, C1, C4). The results demonstrated here, however, suggest that the changes in CBFv are not just a reflection of change in *V*_BP_, and provide further support to autoregulation occurring at the level of the vascular bed, Figure 3. The relative contribution to the hemodynamic response for each paradigm from *V*_CrCP_ and *V*_RAP_ is highly variable, suggesting that activation of different cognitive domains results in different degrees of presumed metabolic and myogenic responses. Nonetheless, all paradigms demonstrate changes in *V*_CrCP_ and *V*_RAP_, presumably reflecting both metabolic and myogenic activation in response to cognitive stimulation.

### Systemic parameters—*V*_BP_, HR, ETCO_2_

The use of subcomponent analysis has a particular advantage to studies using cognitive paradigms, where cognitive tasks have a tendency to produce a marked rise in *V*_BP_ at task initiation due to sympathetic activation (~10 sec) (Moody et al. 2005; Salinet et al. 2013a; van Veen et al. 2015). Certainly in a number of the cognitive paradigms used here, the change in CBFv closely follows that of *V*_BP_, Figures 1, 2. In agreement with this, HR varied significantly between paradigms, indicating a sympathetic response to cognitive testing, Table 2. In a study by Salinet et al., motor active, passive, and imagery paradigms were not distinguishable by CBFv, but could be differentiated according to their presumed myogenic or metabolic responses, providing a more sensitive measure of autoregulation (Salinet et al. 2013a). In both the results reported here, and others (Salinet et al. 2013b), BP modulates the CBFv response over a period of longer than the initial 10 sec. Fundamental to the interpretation of these results, is the observation that if all the changes in CBFv were due to very similar changes in *V*_BP_, then all we are seeing is a passive change in CBF, without the occurrence of metabolic activation, or an autoregulatory response to the significant changes in BP. However, what subcomponent analysis shows is that in the majority of paradigms, *V*_RAP_ is responding to the BP change (Figs. 1, 2) and *V*_CrCP_ is possibly representing the metabolic response. A note of caution is in place however, as these associations cannot be generalized and in other situations it is likely that *V*_RAP_ will also incorporate part of the metabolic response (Panerai et al. 2012b).

In this study, peak percentage change in ETCO_2_ did not differ significantly between paradigms within their respective domain number of paradigms, despite the likelihood of breath‐holding during difficult paradigms, or verbalization in spoken paradigms (Droste et al. 1989). This is in keeping with previous studies where ETCO_2_ remained fairly constant between tasks (Matteis et al. 2009; Salinet et al. 2012a), and contributed relatively little in a multivariate model subcomponent analysis (Panerai et al. 2012a). Nonetheless, the potential effects of ETCO_2_ (Markwalder et al. 1984) are an important consideration for future work involving different cognitive paradigms.

### *V*_CrCP_ and *V*_RAP_

In this analysis, changes in CrCP and RAP have been inverted to represent their effects on the same units as CBFv. Thus, falling CrCP is represented by a rise in its relative subcomponent (*V*_CrCP_), and reflects a positive contribution (increase) to the CBFv response. The same is true of RAP, where rising RAP is represented by a fall in the relative subcomponent (*V*_RAP_) and therefore reflects a negative contribution (decrease) to the CBFv response (Panerai et al. 2005, 2012a).

The results reported in this study give further evidence to the argument for a metabolic component to NVC, and are consistent with a number of studies in the literature (Panerai et al. 2005; Salinet et al. 2013b). In studies by Panerai et al. (2005, 2012a), puzzle and word paradigms were used to activate the nondominant and dominant hemispheres, respectively, with significant differences in the *V*_CrCP_ and *V*_RAP_ responses between paradigms. The RAP + CrCP model was significantly different to the CVRi model (Panerai et al. 2005). *V*_CrCP_ had a predominantly positive change, representing a slower vasodilatory metabolic response to rising oxygen demand, while *V*_RAP_ was predominantly negative, representing the acute myogenic response to rising *V*_BP_ occurring at task initiation (Panerai et al. 2005). Furthermore, they demonstrated a greater metabolic component to the puzzle rather than word paradigm (Panerai et al. 2005, 2012a). In the results reported here, the largest metabolic component was seen with a memory paradigm (A8—learning a name and address). Interestingly, the recall of that name and address produced a much smaller response (C5), perhaps suggesting that learning the information requires greater mental effort than recalling once learnt. The other more metabolically demanding paradigms in this study were spread across the fluency domain (A7), visuospatial (C1 and C2), and language (B2) domains. Many of the attention paradigms produced relatively smaller metabolic responses, and may be less useful in future studies of neuroactivation. Castro et al. (2012) measured subcomponent changes in the PCA during a reading task in healthy volunteers. Measurements were undertaken in varying orthostatic positions and all parameters (*V*_CrCP_, *V*_RAP_ and CVRi) reduced, but responded differently to varying orthostatic conditions (Castro et al. 2012). Variation in *V*_CrCP_ reduced during orthostatic challenge, but increased in CVRi and *V*_RAP_ (Castro et al. 2012). Despite CBFv not changing with orthostatic challenge, the contribution of metabolic processes to NVC were reduced (Castro et al. 2012). Therefore, subcomponent analysis was a more sensitive model of NVC in this study of task activation (Castro et al. 2012). Although TCD measures changes in the MCA, which is one of the larger cerebral arteries, given that it feeds into a network of smaller arterioles which arranged in series, changes occurring at the smaller parenchymal level of arteriole will be transmitted proximally to the MCA, and thus measureable by TCD (Panerai 2003). Certainly, studies demonstrating a distinct correlation between PaCO_2_ and CrCP support the notion that CrCP is a more useful indicator of metabolic changes occurring at the smaller arteriole level (Aaslid et al. 2003; Panerai 2003; Salinet et al. 2013b).

Based on the previous work described above (Panerai et al. 2005), it was expected for all paradigms to produce a predominantly negative *V*_RAP_ response and positive *V*_CrCP_ responses. However, this was not the case. There are a number of potential reasons for the unexpected rises seen in *V*_RAP_. Firstly, on examining Figures 1, 2, changes in *V*_RAP_ reflect those in *V*_BP_, and with significant falls in *V*_BP_, *V*_RAP_ rises correspondingly to normalize CBF in response to *V*_BP_ fluctuation. This is particularly pronounced in paradigms B4, C4 and C1, Figures 1, 2. In agreement with this, a multivariate model by Panerai et al. (2012a) showed *V*_BP_ to be a significant contributor to the *V*_RAP_ response. A similar finding was also seen in an acute stroke population, where *V*_RAP_ fell in response to rising *V*_MAP_ (Salinet et al. 2013b). Furthermore, on visual inspection of individual responses to the same paradigm, the majority of individuals had either a positive or negative RAP response to neuroactivation, suggesting there may be two different types of hemodynamic responses to cognitive stimulation. Close examination of the original responses show a diversity of directional changes in *V*_BP_. The direction of change in *V*_RAP_ was also variable but followed the direction of the *V*_BP_ change. Artifact is unlikely given that no outliers were detected on visual inspection of individual participant data. Finally, the subcomponent analysis used in this study normalizes the percentage change to the preceding 20 sec prior to task initiation, but there is significant variation in the stability of this baseline. In the majority of tasks, there is a negative correlation between peak response in *V*_RAP_ and *V*_BP_, although in practice this is unlikely to reflect the classical “static” correlation, given the inherent time delay in dynamic cerebral autoregulation to respond to changes in MAP (Panerai et al. 2006).

### Hemispheric dominance and metabolic response

The relative contribution of *V*_CrCP_ varied by hemisphere in addition to paradigm, with evidence of lateralization among a number of paradigms. The writing paradigm (B2) produced significant dominant hemisphere lateralization (*P* < 0.005), whereas A6 and B1 all produced significant nondominant hemisphere lateralization (*P* < 0.05) in terms of *V*_CrCP_, which could be reflecting a metabolic response. Of the 20 paradigms presented here, relatively few showed significant lateralization, although previous studies also showed significant lateralization in *V*_RAP_, but not *V*_CrCP_ response (Panerai et al. 2005, 2012a). The atypical V_RAP_ response seen in a number of paradigms, was subject to lateralization in the A1, B4, B5, and C1 paradigms, where a typical response was seen in the contralateral hemisphere. A1, B4, and B5 demonstrated dominant side lateralization of the atypical response and C1, nondominant, but we are not clear at present why there are hemispheric differences in the *V*_RAP_ response for these paradigms.

### Clinical studies of subcomponent analysis

Few studies have examined subcomponent analysis in pathological states, and none in cognitive impairment to date. Castro et al. (2014) analyzed the subcomponent changes in patients with autonomic failure (AF) compared to healthy controls during the Valsalva maneuver. AF patients had a more pronounced decline in CBFv compared to controls, whereas *V*_CrCP_ and CVRi increased to a similar extent in both groups (Castro et al. 2014). The *V*_RAP_ response was greater in AF, possibly due to compensation, but the results suggest the CrCP + RAP model allows a better understanding of the autoregulatory process than CVRi in this population (Castro et al. 2014). In contrast to this study, *V*_RAP_ was a more useful indicator of the mechanisms underlying the CBFv changes (Castro et al. 2014), whereas here we found *V*_CrCP_ to be a more useful measure of the metabolic cognitive load of different paradigms. In this study, changes in *V*_RAP_ were variable, and mainly reflected the changes in *V*_BP_, suggesting it may be a less reliable method of measuring NVC in task activation.

Maggio et al. (2013) used CO_2_ inhalation during a passive motor paradigm to induce an impaired dCA state in healthy controls, to model that of acute stroke. CBFv, BP, and ETCO_2_ were all significantly higher, but *V*_CrCP_ was significantly lower during CO_2_ inhalation/impaired regulation, suggesting the metabolic response is affected (Maggio et al. 2013). However, significant differences can occur between motor and mental paradigms, given that cognitive paradigms are more mentally challenging and can require greater sympathetic activation (Salinet et al. 2013a). Therefore, modeling the subcomponent changes under pathological conditions using cognitive paradigms would provide more information on NVC in response to task activation.

In a study of pre‐eclamptic women, during the breath‐holding maneuver, changes in CBFv, MAP, V_CrCP_, and ETCO_2_ were similar between healthy and pre‐eclamptic women (van Veen et al. 2015). However, CVRi and *V*_RAP_ failed to rise to levels seen in healthy controls at initiation of the breath‐holding maneuver (van Veen et al. 2015). These results were similar to those seen in acute stroke patients, suggesting a failure of myogenic autoregulation (Salinet et al. 2013b; van Veen et al. 2015). In these studies, *V*_CrCP_ remained similar between groups, suggesting metabolic regulation remains relatively intact in these conditions (Salinet et al. 2013b; van Veen et al. 2015).

The disruption of cerebral autoregulation is now well documented in AzD, VaD, and at the precursor stage of MCI (Keage et al. 2012; Sabayan et al. 2012), but it is not known if this is predominantly a failure of metabolic or myogenic regulation. In both acute stroke and pre‐eclamptic patients (Salinet et al. 2013b; van Veen et al. 2015), the metabolic pathway remained intact, however, functional decoupling in cognitive impairment may differ significantly as a result of differing disease processes.

### Study limitations

There are a number of limitations which merit further discussion. First, the use of TCD to measure CBFv rests on the assumption that the diameter of the measured vessel remains relatively constant, which has been demonstrated with small fluctuations in ETCO_2_ (± 1 kPa), but does change over larger fluctuations in ETCO_2_ (>2 kPa) (van Beek et al. 2008; Verbree et al. 2014; Mikhail Kellawan et al. 2016). Secondly, subcomponent analysis is limited by poor signal‐to‐noise ratio in estimates of *V*_RAP_ and *V*_CrCP_, which can be improved by repeated testing (Panerai et al. 2012a; Salinet et al. 2013b). This is difficult, however, in studies using cognitive task activation, which risk fatigue and accommodated responses to repeat stimulation (Goldberg et al. 2015). In a previous study, the methods used here have been shown to be robust (Panerai et al. 2011). Thirdly, while we report here that myogenic and metabolic responses are broadly represented by *V*_RAP_ and *V*_CrCP_, respectively, there could be a considerable overlap between the two and they are not mutually exclusive (Panerai et al. 2005). Additionally, if the baseline values used for normalization are relatively low, this could give the appearance of a rising *V*_RAP_, when in fact the baseline showed large variability or was not representative for that individual. The hemispheric difference in *V*_RAP_ response among a number of paradigms could be a result of differing baseline values of CBFv and *V*_RAP_ in each hemisphere, thus the normalization required by SCA can increase differences. The other possibility is the ‘purity’ of the myogenic association. In many cases it is possible that *V*_RAP_ might also be sensitive to metabolic influences, including differential changes in ETCO_2_ and MAP. Fourthly, in this study, only the MCA was insonated, and not the posterior (PCA) or anterior (ACA) cerebral arteries. This limits the analysis, both to the region supplied by the MCA (approximately 80% of the cortex) (van Beek et al. 2008), and the ability to perform detailed spatial mapping of subcomponent responses. A number of the paradigms used in this study would activate areas supplied by the ACA and PCA (i.e., visuospatial paradigms), and so the responses seen here may not accurately reflect their full activation profiles. Fifthly, the reproducibility of CBFv responses to task activation is an important consideration in the clinical application of this modality, but few studies have investigated this, and those that have yielded variable results (Stroobant and Vingerhoets 2001; Vingerhoets and Stroobant 2002; Salinet et al. 2012b; Beishon et al. 2017a). Sixthly, previous studies have used multivariate modeling to adjust for and define the relative contributions of each subcomponent to the CBFv response (Panerai et al. 2012a; Salinet et al. 2013a). Furthermore, the AUC V_CrCP_ analysis may not be fully accurate given that a number of participants did not return to baseline, despite a 30‐sec rest period between tasks. Therefore, future studies should consider longer rest periods between cognitive tasks. Finally, the population studied here was relatively young, predominantly right hand dominant, and Caucasian females, limiting the generalizability of the results.

### Further work

Future studies should consider the use of more diverse groups with greater representation of the general population. The results demonstrated here also warrant further investigation in patient populations, specifically, the integrity of the metabolic and myogenic processes underpinning autoregulation and how they are affected by dementing disease processes. This raises the questions of how early the disease abnormalities in these components can be detected, and whether it varies according to dementia subtype (i.e., AzD, VaD). Furthermore, whether CrCP and RAP are more sensitive discriminators of hemodynamic dysregulation, and the potential validity of these markers in distinguishing early cognitive impairment from normal aging. Additionally, the reproducibility of CBFv responses to motor paradigms has been demonstrated (Salinet et al. 2012b), but not in a subcomponent analysis. CBFv responses to task activation can exhibit significant within subject variability, therefore, the reproducibility of subcomponent analysis requires validation using the ACE‐III, in both a healthy control population and a cognitively impaired population. The use of near‐infrared spectroscopy could also be considered as an adjunct to TCD, allowing for enhanced spatial discrimination of task activated responses, and localization to the level of the vascular tree at which these are occurring (Phillips et al. 2016).

## Conclusions

Neuroactivation with cognitive tasks results in changes in cerebral hemodynamics that can be detected at the level of myogenic and metabolic responses using functional TCD, providing a more detailed model of NVC in cognitive stimulation. This technique now requires further investigation into the integrity of subcomponent responses in a cognitively impaired population, and the ability to distinguish healthy controls from those with cognitive impairment, which may be more sensitive than measures of CBFv alone.

## Conflicts of Interest

None to declare.
